# A Critical Need for Advanced Practice Nurse Education in Sub‐Saharan Africa and Lessons Learned From the COVID‐19 Pandemic: A Discursive Review

**DOI:** 10.1002/nop2.70079

**Published:** 2024-12-22

**Authors:** Jacqueline Itambo, Brenda Owusu, Danielle McCamey, Diana‐Lyn Baptiste

**Affiliations:** ^1^ School of Nursing Johns Hopkins University Baltimore Maryland USA; ^2^ School of Nursing Colorado State University Pueblo Colorado USA; ^3^ School of Nursing and Health Studies University of Miami Coral Gables Florida USA

**Keywords:** advanced practice registered nurse, COVID‐19 pandemic, health disparities, nurse practitioner, nursing education, sub‐Saharan Africa

## Abstract

**Aim:**

To discuss the need to expand advanced practice nurse education in Sub‐Saharan Africa as a strategic response to the impact of the COVID‐19 pandemic.

**Design:**

Discursive paper.

**Method:**

Searching international literature in PubMed, CINAHL and Google Scholar databases, we explored the impact of COVID‐19 in Sub‐Saharan Africa and the advanced practice nurse role and education in the pandemic response.

**Discussion:**

The COVID‐19 pandemic served as a barrier to healthcare access and disrupted routine immunizations and care, reduced facility‐based healthcare services, increased disease burden and mortality, strain and is expected to worsen the healthcare workforce shortage in Sub‐Saharan Africa. The COVID‐19 pandemic has further emphasised the necessity to strengthen advanced practice nurse education in Sub‐Saharan Africa.

**Conclusion:**

This discursive paper underscores the critical role of advanced practice nurse education in Sub‐Saharan Africa as a proactive strategy to strengthen the healthcare workforce and systems. The lessons learned from the COVID‐19 pandemic highlight the urgency to invest in advanced practice nurse education and training programmes in the region.

**Patient or Public Contribution:**

There was no patient or public involvement in the design or drafting of this discursive paper. The authors reviewed the literature to develop a discussion about advanced nursing practice.

## 1 Introduction

Sub‐Saharan African (SSA) countries are defined as those countries south of Sahara Desert home to 1.18 billion people with 34.9% of the population living in poverty (The World Bank 2021). The Sub‐Saharan region comprises 46 of the 55 countries in Africa which include Botswana, Ghana, Malawi, Nigeria, South Africa, Tanzania, Egypt, Uganda, Malawi, Zimbabwe, Cameroon and Ethiopia. The SSA countries carry 24% of the global disease burden with less than 1% global expenditure allocated to health (Spearman and Sonderup 2015). Subsequently, most of these countries experience major health disparities in health care access, healthcare workforce shortage and inadequate healthcare infrastructure which were further exacerbated by the COVID‐19 pandemic (Okoi and Bwawa 2020). The impact of COVID‐19 on health, social, economy and daily lives, is unprecedented in modern times and has changed the landscape of the advanced practice nurse role in improving health care access and healthcare systems.

The National Council of State Boards of Nursing (NCSBN) defined Advanced Practice Registered Nurses (APRNs) as nurses prepared at the master's or post master's level in a specific clinical role or patient population (NCSBN 2022). APRNs can take on the titles of clinical nurse specialist, certified nurse practitioner, certified registered nurse anaesthetist, or certified nurse‐midwife (NCSBN 2022). They are crucial in addressing the shortage of physicians, improve health care access and provide cost‐effective care, which is why some countries have expanded APRNs role to meet these challenges (Nardi and Diallo 2014). The COVID‐19 pandemic highlighted the importance of APRNs and their essential role in preparedness to respond to urgent healthcare needs. However, the implementation of advanced practice nurse education programmes in SSA countries has been sparse, with some countries not having APRN education programmes at all (Christmals and Armstrong 2019).

## 2 Background

The APRN role was first started in the United States in 1965 to address the physician shortage (Brennan 2020). In the United Kingdom, the advanced nurse practice role developed rapidly in the 1990s to meet the fast‐growing health needs of the society (Swaby, Reynolds, and Mortimore 2022). Since its inception, advanced nurse education and practice have been hailed as successful models in health education, health promotion and disease prevention with countries such as the United Kingdom, Canada and Australia reporting significant gains in healthcare quality and access (Scanlon et al. 2015). Some countries in SSA such as Botswana, Ghana, Malawi, Nigeria, South Africa and Tanzania have attempted to establish advanced practice nurse education. However, the lack of legislation and formal curriculum to guide the development of successful APRN programmes have been cited as major challenges in the region (Christmals and Armstrong 2020).

The World Health Organization (WHO) strongly advocates for primary care to improve access to improve quality of care, prevent diseases, save lives and increase healthy life expectancy in SSA (World Health Organization 2021a). APRNs have the potential to reduce health inequalities and provide healthcare across the lifespan in clinical settings of acute care, primary care and clinical specialty areas. APRNs have made a significant impact in health delivery, where they attend to individuals irrespective of their social class, location, demographics, or background (Rantz et al. 2018).

Over several decades, SSA countries have experienced multiple outbreaks of infectious diseases with notable examples including Ebola virus outbreak (Ihekweazu and Agogo 2020). Additionally, these countries bear a heavy disease burden, particularly in relation to HIV/AIDS, Tuberculosis (TB) and malaria. SSA has 26 million people living with HIV and 5000 new HIV infections per day reported in these countries (Mhango, Chitungo, and Dzinamarira 2020). Despite TB being a curable disease, SSA countries continue to face high infection rates with 10 million reported infected in 2019 (Velavan et al. 2021). Given the significant disease burden, SSA's fragile health care systems are at substantial risk of collapse, particularly when faced with added weight of a pandemic such as COVID‐19. The focus of this discursive paper is to discuss the need to expand advanced practice nurse education in Sub‐Saharan Africa as a strategic response to the impact of the COVID‐19 pandemic.

## 3 Method

### 3.1 Data Sources

Searching international literature in PubMed, EBSCOhost and Google Scholar databases, we explored the impact of COVID‐19 in Sub‐Saharan Africa and the advanced practice nurse role and education in the pandemic response.

## 4 Discussion

**FIGURE 1:**
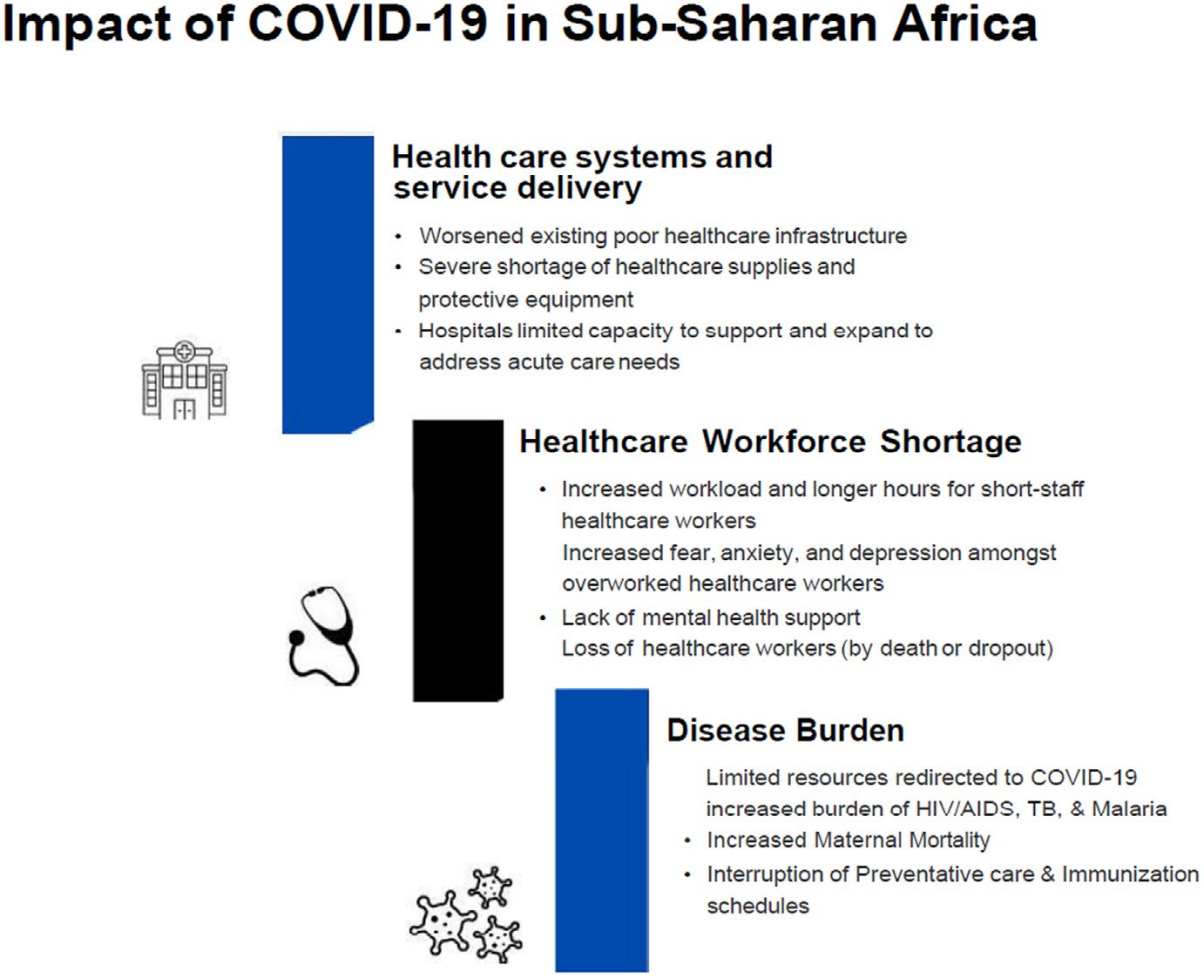
Impact of COVID‐19 in Sub‐Saharan Africa. The figure outlines how the pandemic strained healthcare systems through worsened infrastructure, supply shortages, and overwhelmed hospitals. It highlights workforce challenges including increased workloads and mental health issues. Additionally, it shows how diverted resources increased the burden of diseases like HIV/AIDS, TB, and malaria, disrupting immunisation schedules, and worsened maternal mortality.

Figure 1 illustrates the impact of the COVID‐19 pandemic on the disease burden, healthcare workforce shortage and healthcare systems and delivery of healthcare services in SSA. Additionally, we discuss strategies for developing APRN educational programmes through global partnerships.

### 4.1 Impact on the Disease Burden in SSA

In SSA, COVID‐19 first was reported in Egypt in February 2020 (Uwaezuoke 2020), and in the following months, cases started being reported from various countries in SSA. SSA cases remained steadily low probably due to widespread under testing and underreporting stemming from weak healthcare systems and poor surveillance systems (Uwaezuoke 2020). While reported cases of COVID‐19 active disease, recoveries and mortality rates across SSA remained low, the disease burden of HIV/AIDS, TB and malaria increased (Inzaule et al. 2021; World Health Organization 2022). This was an indication that COVID‐19 increased the disease burden in SSA, first by redirection of already‐strained healthcare resources to COVID‐19 containment, and second by interruption of health care measures already in place to address the major disease burdens.

Countries in SSA had to redirect their limited healthcare resources to fight and manage COVID‐19 which limited healthcare access for non‐COVID‐19 diseases (Inzaule et al. 2021; Wallace et al. 2020). The direct impact of the COVID‐19 pandemic on increasing the burden of other chronic diseases in SSA is outlined in recent literature. A study done in Uganda from January 2019 through March 2020, showed that reduced healthcare access led to increased maternal mortality, reduced facility deliveries and reduced HIV/AIDS and malaria treatments (Bell et al. 2020). Essential services such as immunisation schedules and preventive care were interrupted as people abstained from visiting healthcare facilities or primary care doctors (Velavan et al. 2021; Tessema et al. 2021). In South Africa, Malawi, Zimbabwe and Uganda, it was projected that HIV‐related fatalities during the COVID‐19 pandemic were likely to increase due to HIV treatment interruptions (Velavan et al. 2021). Fear of contracting COVID‐19 in public areas and strict lockdowns meant people were not able to access care and continue with treatment regimens.

The WHO world report in 2020 on malaria acknowledged the disruption of essential healthcare services such as immunisation, facility‐based treatment of non‐communicable diseases, antenatal care and family planning, among others (World Health Organization 2021b). To support WHO malaria report, countries like Cameroon reported increment in malaria cases and deaths during the COVID‐19 pandemic and Zimbabwe reported shortage of antimalarial drugs and a lack of access to healthcare (Velavan et al. 2021). Studies showed that TB infection rates dropped in 2020 compared to 2019 to show that there was significant disruption in treatment and tracking of new cases (Velavan et al. 2021).

To reduce the disease burden, increase health care access and better manage pandemics, SSA countries should implement advanced practice nurse education and training programmes. The nursing workforce in SSA which is about 37% (Ahmat et al. 2022) could serve as a pipeline for advance practice nurse graduates. APRNs provide care for a diverse patient population and are likely to serve in rural and medically underserved areas (Stucky, Brown, and Stucky 2020) where most of the African population live (Christmals and Armstrong 2019).

### 4.2 Impact on Healthcare Workforce Shortage in SSA

In SSA, there is a scarcity of physicians, nurses and advanced practice providers across all clinical areas including primary care and inpatient care settings with ratios of 2.3 healthcare workers per 1000 population (Dyer 2020; Tessema et al. 2021). The healthcare worker shortage compounded by the COVID‐19 management crisis meant increased workload and longer working hours for healthcare workers. Hospitals struggled with a shortage of healthcare workers specialist in critical care and infectious diseases (Tessema et al. 2021; Barasa, Ouma, and Okiro 2020). These healthcare shortages were further worsened by a lack of support for healthcare workers dealing with COVID‐19 management (Sagaon‐Teyssier et al. 2020).

A systematic review by Okpua et al. (2021), showed that frontline COVID‐19 healthcare workers in Africa and Asia suffered symptoms consistent with psychological and physical impacts due to the pandemic. These adverse effects worsened as the healthcare shortage worsened and workload increased for the remaining frontline teams. In SSA, frontline workers had no specific training to manage COVID‐19 and lacked personal protective equipment which often resulted in fear, anxiety and depression among healthcare workers (Okpua et al. 2021; Tessema et al. 2021). Healthcare workers themselves needed the training to build confidence and competency to manage the crisis, and mental support, something that many SSA health systems were unable to provide. Widespread fear, anxiety, burnout, infections and healthcare workers' COVID‐19 related deaths led to increased dropout which further worsened the healthcare workforce shortage in SSA (Nchasi et al. 2022).

High‐income countries used APRNs to mitigate healthcare worker shortages created by COVID‐19. In the United States, emergency regulations were passed to expand the practice role of APRNs and recall retired APRNs to rejoin the healthcare workforce and remain in the frontline of the pandemic (Stucky, Brown, and Stucky 2020; Diez‐Sampedro et al. 2020). Based on gaps identified in response to the COVID‐19 pandemic in SSA, the WHO has provided eleven strategic response pillars (World Health Organization 2021b) to manage COVID‐19 and future pandemics in SSA. One of the pillar's objectives is to maintain essential health service delivery, mitigate the risk of health system collapse, contribute to long‐term health system resilience and progress toward universal health coverage (World Health Organization 2021a). Training and capacity building for healthcare workers was identified as a key component for meeting the pillar's objectives. To achieve the objectives of this pillar, SSA countries should consider investing in APRN education and training.

### 4.3 Impact on Health Care Systems and Delivery of Healthcare Services

COVID‐19 arrived and landed on a weak, overburdened, fragile healthcare system in SSA (Paintsil 2020; Tessema et al. 2021). It is well reported that SSA struggled with a limited healthcare workforce, limited intensive care units, poorly equipped hospitals and poor healthcare infrastructure which worsened with the COVID‐19 pandemic (Tessema et al. 2021). A good example of resources diversion was in Ethiopia where TB treatment facilities were converted to COVID‐19 isolation and treatment centres (Mohammed et al. 2020).

Healthcare centres struggled with resources and care delivery under the pressure of the COVID‐19 pandemic and were reported to be in severe short supply of protective equipment and increasingly becoming the centres of infection and transmission of the virus to both healthcare workers and patients (Bajaria and Abdul 2020; Desalegn et al. 2021). Hospitals struggled with limited capacity and inability to quickly expand to address much‐needed acute and critical care settings to treat COVID‐19 patients (Tessema et al. 2021; Barasa, Ouma, and Okiro 2020). In SSA, communities live in crowded areas, share amenities, and have no running water making it difficult for governments and health systems to enforce COVID‐19 containment measures such as social distancing, hand washing, self‐isolation at home and contact tracing. COVID‐19 containment measures were mostly focused on urban areas and rural areas were left out due to limited resources, scarcity of healthcare workers, limited healthcare centres and limited logistics planners.

In countries with well‐established APRN programmes, APRN providers stepped in to meet the challenges of healthcare delivery and improved access during the pandemic. In the United States, APRNs were deployed and provided care in emergency rooms, intensive care units and primary and public health care settings (Proulx 2020). Changes in legislation enabled APRNs to provide care via Telemedicine (Proulx 2020; Stucky, Brown, and Stucky 2020). APRNs proved to be innovative in creating effective COVID‐19 management and delivery of care. Emory Healthcare, a large health system in the United States, implemented APRN‐led patient care models based on skill levels, to help handle the influx of the acutely ill and critical care of hospitalised COVID‐19 patients. High‐income countries and organisations such as the World Bank and the WHO have stepped in to support SSA to combat COVID‐19 with equipment and financial support (Mezue et al. 2020). This effort is not sustainable unless there is a healthcare workforce equipped with the right advanced practice skills and knowledge to manage the health care delivery system in SSA.

One significant barrier to the development of advanced practice roles in SSA is the lack of educational infrastructure to support APRN training. Many countries in the region lack the advanced nursing education programmes and faculty needed to train APRNs. Moreover, the absence of regulatory frameworks to formalise and licence APRNs has stalled progress in expanding their role in healthcare systems (Oleribe et al. 2019; Uwaezuoke 2020). Additionally, financial constraints and resource limitations make it difficult to establish and maintain APRN programmes, as healthcare budgets are often directed toward basic health services. Cultural and professional hierarchies also contribute to resistance from the medical community, where physician‐led models dominate and APRN roles may be underutilised (Oleribe et al. 2019; Uwaezuoke 2020). Without international collaboration and significant investments in education, policy reforms and infrastructure, the growth of the APRN workforce will remain a challenge in SSA.

### 4.4 Influence of Global Partnerships to Increase APRN Education Programmes

Increasing APRN programmes in SSA will require a multi‐faceted approach to address education, funding, infrastructure and policy (Wheeler et al. 2022). Collaborating with international institutions to establish exchange programmes enhances faculty expertise, thereby equipping more educators to support APRN programmes in SSA. International Universities can provide expertise to develop and standardise APRN curricula tailored to address healthcare needs in SSA (Wheeler et al. 2022). Online APRN programmes developed in collaboration with international universities will help provide accessible education and increase the supply of APRN providers in remote and underserved areas of SSA (Wheeler et al. 2022). International universities in collaboration with nursing international organisations such as the International Council of Nurses, Global Nurses United, WHO and the International Organization of African Nurses can work with African governments to advocate for funding of APRN education, establish a regulatory framework and scope of practice, and develop a system for licensing and accreditation (Oleribe et al. 2019; Wheeler et al. 2022). By implementing these strategies and many others, SSA countries can significantly expand APRN educational programmes, address healthcare provider shortage and improve preparedness to combat future pandemics.

## 5 Conclusion

The impact of COVID‐19 on health and health systems in SSA is evidence that future pandemics have the potential to disrupt routine immunizations and care, reduce facility‐based healthcare services, increase disease burden, increase mortality from other diseases and reduce the healthcare workforce. Investing in APRN education and training in SSA is crucial for strengthening healthcare systems, enhancing preparedness for future pandemics and improving the healthcare outcomes of individuals in the region. It is imperative that policymakers, healthcare institutions and stakeholders prioritise and support the development and implementation of APRN educational programmes in Sub‐Saharan Africa to address the healthcare challenges posed by COVID‐19 pandemic and future health crises.

## Conflicts of Interest

The authors declare no conflicts of interest.

## Data Availability

Data sharing is not applicable to this article as no new data were created or analyzed in this study.
